# Genetic Testing of Korean Familial Hypercholesterolemia Using Whole-Exome Sequencing

**DOI:** 10.1371/journal.pone.0126706

**Published:** 2015-05-11

**Authors:** Soo Min Han, Byungjin Hwang, Tae-gun Park, Do-Il Kim, Moo-Yong Rhee, Byoung-Kwon Lee, Young Keun Ahn, Byung Ryul Cho, Jeongtaek Woo, Seung-Ho Hur, Jin-Ok Jeong, Sungha Park, Yangsoo Jang, Min Goo Lee, Duhee Bang, Ji Hyun Lee, Sang-Hak Lee

**Affiliations:** 1 Department of Pharmacology, Brain Korea 21 PLUS Project for Medical Sciences, Severance Biomedical Science Institute, Yonsei University College of Medicine, Seoul, Korea; 2 Department of Chemistry, Yonsei University, Seoul, Korea; 3 Cardiology Division, Department of Internal Medicine, Haeundae Paik Hospital, Inje University College of Medicine, Busan, Korea; 4 Cardiovascular Center, Dongguk University Ilsan Hospital, Goyang, Korea; 5 Cardiology Division, Department of Internal Medicine, Gangnam Severance Hospital, Yonsei University College of Medicine, Seoul, Korea; 6 Heart Center of Chonnam National University Hospital, Gwangju, Korea; 7 Cardiology Division, Department of Internal Medicine, Kangwon National University Hospital, Kangwon National University College of Medicine, Chunchon, Korea; 8 Endocrinology Division, Department of Internal Medicine, Kyunghee University School of Medicine, Seoul, Korea; 9 Cardiology Division, Department of Internal Medicine, Keimyung University Dongsan Medical Center, Daegu, Korea; 10 Cardiology Division, Department of Internal Medicine, School of Medicine, Chungnam National University, Chungnam National University Hospital, Daejeon, Korea; 11 Cardiology Division, Department of Internal Medicine, Severance Cardiovascular Hospital, Seoul, Korea; 12 Cardiovascular Research Institute and Cardiovascular Genome Center, Yonsei University Health System, Seoul, Korea; 13 Department of Oral Biology, College of Dentistry, Yonsei University, Seoul, Korea; Odense University hospital, DENMARK

## Abstract

Familial hypercholesterolemia (FH) is a genetic disorder with an increased risk of early-onset coronary artery disease. Although some clinically diagnosed FH cases are caused by mutations in *LDLR*, *APOB*, or *PCSK9*, mutation detection rates and profiles can vary across ethnic groups. In this study, we aimed to provide insight into the spectrum of FH-causing mutations in Koreans. Among 136 patients referred for FH, 69 who met Simon Broome criteria with definite family history were enrolled. By whole-exome sequencing (WES) analysis, we confirmed that the 3 known FH-related genes accounted for genetic causes in 23 patients (33.3%). A substantial portion of the mutations (19 of 23 patients, 82.6%) resulted from 17 mutations and 2 copy number deletions in *LDLR* gene. Two mutations each in the *APOB* and *PCSK9* genes were verified. Of these anomalies, two frameshift deletions in *LDLR* and one mutation in *PCSK9 *were identified as novel causative mutations. In particular, one novel mutation and copy number deletion were validated by co-segregation in their relatives. This study confirmed the utility of genetic diagnosis of FH through WES.

## Introduction

Familial hypercholesterolemia (FH) is a genetic disorder characterized by high levels of serum low-density lipoprotein cholesterol (LDL-C) and an increased risk of premature coronary artery disease. It is commonly caused by loss-of-function mutations in *LDLR*, mutations in *APOB*, or less-frequent gain-of-function mutations within *PCSK9*. FH is clinically diagnosed based on serum cholesterol levels, physical examinations, and family history. Clinical diagnostic criteria and guidelines have been developed by experts in this field, though controversies remain. [1] The confirmation of mutations through DNA testing can allow for targeted family screening. It has also been proven to be highly efficient for patient identification. [2]

To this date, several genetic techniques are available for the diagnosis of FH. Assay systems that are designed for the most common mutations have typically been used. [3] However, genetic screening through conventional molecular diagnostic techniques has limitations for effective FH diagnosis due to the wide variety of types and locations of mutations in known genes [4], as well as the existence of undiscovered or potential FH-causing genes. [5–7] Next-generation sequencing (NGS) is a powerful tool for discovering genetic mutations in large genomic regions and novel disease-related genes. Several studies have demonstrated the utility of NGS in the diagnosis of FH. [8–10]

In the present study, we utilized whole-exome sequencing (WES) in Korean FH patients to provide insight into the spectrum of mutations causing FH. We also investigated clinical phenotypes in FH patients with and without mutations.

## Materials and Methods

### Patient enrollment

Patients with FH and their family members were recruited at nine sites in Korea. Each center’s Institutional Review Board (IRB) approved the protocol. Initially 136 suspicious patients were referred for FH, and among them, 66 were excluded due to uncertain family history. One patient was a family member of a formerly enrolled proband and was also excluded. Ultimately, the exomes of 69 FH patients were analyzed. All subjects or their representatives gave written informed consent. All clinical investigations were conducted in accordance with the principles of the Declaration of Helsinki. This study was approved by the IRB of Severance Hospital at Yonsei University College of Medicine in Korea (IRB No.: 4-2008-0267).

### Clinical and laboratory Assessment

The clinical diagnosis of FH is based on the Simon Broome criteria of the UK. [11] Specifically, the criteria for definite FH were defined as total cholesterol (TC) > 290 mg/dL or LDL-C > 190 mg/dL the presence of tendon xanthomas in either the patient or relatives, or genetic evidence of mutations in *LDLR*, *APOB* or *PCSK9*. The criteria for possible FH were defined as the cholesterol levels described above, with the addition of a family history of premature myocardial infarction or raised cholesterol > 290 mg/dL. At the time of enrollment, each patient underwent medical history interviews, comprehensive physical examinations, and laboratory assessments.

TC, triglycerides (TGs), high-density lipoprotein cholesterol (HDL-C), and LDL-C were measured in all subjects. Patients fasted and avoided alcohol and smoking for at least 12 hours prior to blood sampling. Samples were analyzed within 4 hours by laboratories that were certified by the Korean Society of Laboratory Medicine.

### Control Group

Exome sequencing data of Koreans without the FH phenotype were used as controls (n = 390). We collected 95 raw exome data sets from the National Biobank of Korea. The other 295 exome data sets were generated at Yonsei University. All control data sets were processed under the same analysis pipeline that processed the FH patients’ exomes. The control group had no conditions that were known to affect plasma lipid levels.

### WES and data analysis

Exome sequencing was performed by the Agilent SureSelect Enrichment System, according to the manufacturer's protocol. Due to differences in collection times, the SureSelect All Exon 50Mb kit was used in 44 patients for the first set of exome analysis, whereas the SureSelect All Exon V4+UTRs kit was used in 25 patients for the second set of exome analysis. Sequencing of exome was performed on Illumina HiSeq2000/2500 platforms with 101-bp and 150-bp paired-end sequencing for each set. The sequencing paired reads were mapped to the reference genome, NCBI Build 37 (hg19), by Novoalign (v2.07.18). Primarily aligned reads were re-aligned locally near indels and were further processed with recalibrated quality scores, using the Genome Analysis Toolkit (v2.3.6). [12, 13] PCR duplicates were removed by Picard (v1.6.7). Sequencing data are accessible at Sequence Read Archive (http://trace.ncbi.nlm.nih.gov/Traces/sra/; accession number SRA: SRA245003). Variant calls were conducted using the Unified GATK Genotyper (v2.3.6) and retained only if being called with a minimum of 8× coverage. The summary of the overall exome sequencing data for each set is shown in S1 Table. Additionally, annotations and functional effect predictions for single nucleotide variants (SNVs) were performed by PolyPhen-2 (v2.2.2), [14] SIFT, [15] and ANNOVAR. [16] Small indels were annotated by ANNOVAR. Additionally, we rescreened total variants for splicing sites and variable promoter regions, which were likely to be missed by conventional annotation programs. For splicing site variants, we screened ±50bp of exons from all transcript isoforms that were downloaded from the UCSC Table Browser. We screened for known pathogenic variants in the promoters of *LDLR*, *APOB*, and *PCSK9* by referring to the public database (The Human Gene Mutation Database, HGMD). Finally, we inferred changes in copy number from exome-sequenced reads by the CoNIFER (COpy Number Inference From Exome Reads) algorithm, [17] which utilizes singular value decomposition normalization. Copy number variants were validated by the TaqMan Copy Number Assay (S2 Table).

### Pathogenicity prediction and mutation validation

Pathogenic mutations in *LDLR*, *APOB*, and *PCSK9* were classified based on previously reported causal variants from public databases (LOVD-LDLR, LSBD-UMD-LDLR, LOVD2-LDLR, and HGMD). The pathogenicity of unreported variants in *LDLR*, *APOB*, and *PCSK9* were predicted based on the 1) deleterious effects of amino acid changes and evolutionary conservation, as predicted by Polyphen-2 and SIFT algorithms, 2) frequency in 390 Korean controls and public databases (1000 Genomes Project, dbSNP135, and NHLBI GO Exome Sequencing Project [ESP]), and 3) co-segregation of identical variants in family members, if available. The predicted deleterious mutations that were absent in controls yet present in affected family members were classified as novel pathogenic mutations. All variants classified as known or novel pathogenic mutations in the three FH genes were confirmed by Sanger sequencing.

## Results

### Clinical characteristics of study subjects

The characteristics of enrolled patients are shown in Table 1. We found that 23 of 69 patients harbored FH-linked mutations in *LDLR*, *APOB*, or *PCSK9*. We defined mutation-positive patients by confirming known and novel genetic aberrations in these three genes. All other patients were classified as mutation negative. Compared to mutation-negative patients, mutation-positive patients were more frequently males with higher LDL-C levels (223±39 mg/dL vs. 246±41 mg/dL; *p* = 0.02). In addition, mutation-positive patients tended to be younger than mutation-negative patients. However, there was no difference in history of coronary artery disease between the two groups.

**Table 1:** Clinical characteristics of enrolled familial hypercholesterolemia patients.

	Total (n = 69)	Mutation (-) (n = 46)	Mutation (+) (n = 23)	P*
Age, years	54±13	56±12	50±14	0.09
Gender, Male	29 (42)	15 (33)	14 (61)	0.03
Medical history				
Hypertension	33 (48)	23 (50)	10 (43)	0.61
Diabetes mellitus	6 (9)	4 (9)	2 (9)	1.00
Coronary artery disease	25 (36)	17 (37)	8 (35)	0.86
Smoking	15 (22)	10 (22)	5 (22)	1.00
Family history				
Myocardial infarction	38 (55)	23 (50)	15 (65)	0.23
Total cholesterol>290 mg/dL	43 (61)	26 (57)	16 (70)	0.30
Clinical classification of FH				
Definite	17 (25)	10 (22)	7 (30)	0.43
Possible	52 (75)	36 (78)	16 (70)	
Physical findings				
Body mass index, kg/m^2^	25.0±3.6	25.1±3.5	25.0±3.9	0.96
Xanthoma	17 (25)	12 (26)	5 (22)	0.69
Laboratory values				
Total cholesterol, mg/dL	316±48	308±50	330±43	0.08
Triglyceride, mg/dL	174±85	184±94	155±60	0.19
HDL-cholesterol, mg/dL	47.4±11.2	48.5±10.9	45.2±11.8	0.26
LDL-cholesterol, mg/dL	230±41	223±39	246±41	0.02

*Chi-square test or t-test was used where appropriate.

Values are mean ± standard deviation or n (%).

HDL: high-density lipoprotein; LDL: low-density lipoprotein; Mutation (-): No known or novel pathogenic mutations in three FH-linked genes (*LDLR*, *APOB*, *PCSK9*); Mutation (+): Known or novel pathogenic mutations in *LDLR*, *APOB*, or *PCSK9*.

### Detection and validation of known mutations in three FH-linked genes

**Fig 1:**
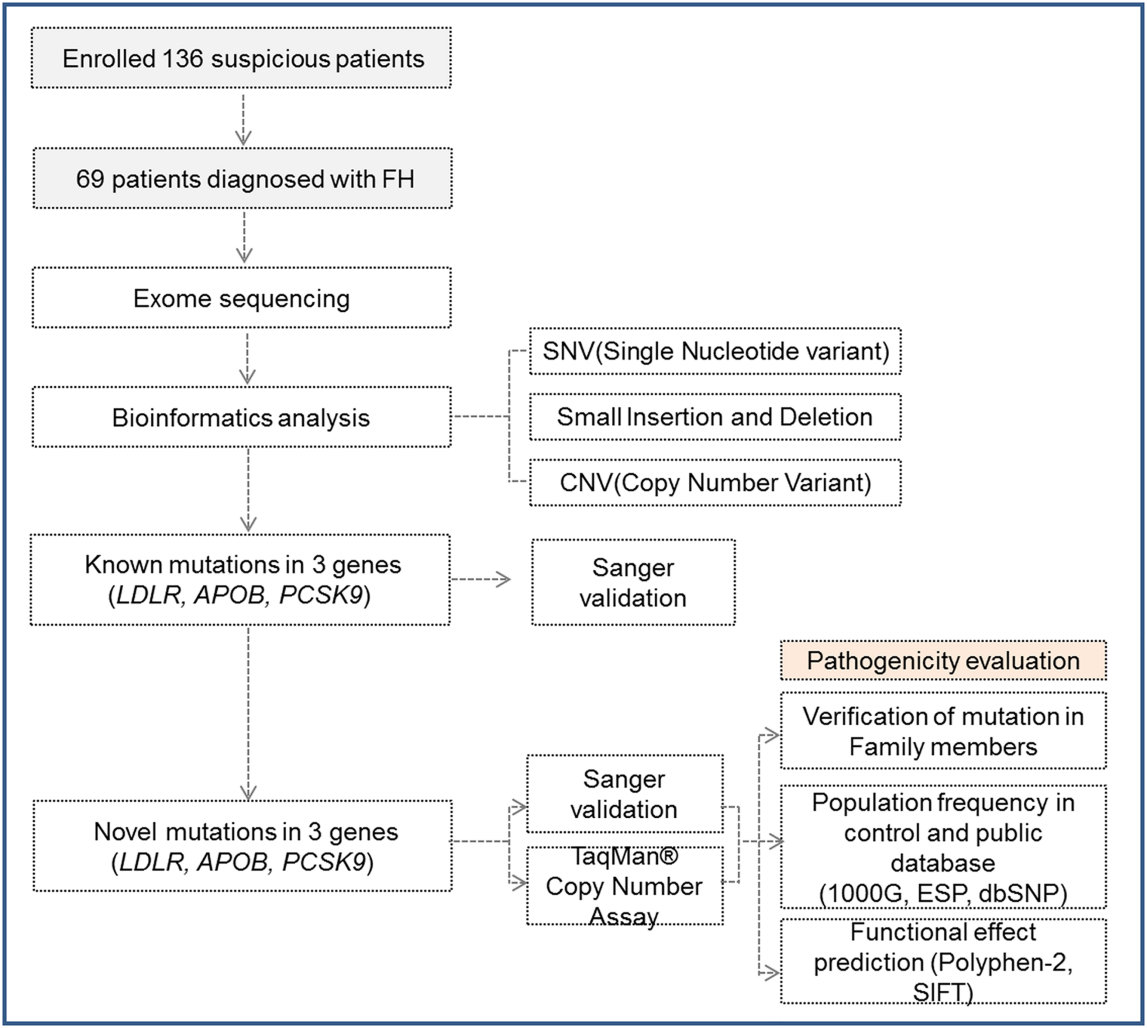
Exome sequencing analysis of familial hypercholesterolemia (FH). The steps for identifying FH-causing variants in three genes are shown, in addition to the subsequent genetic analyses of whole-exome sequencing data that led to the identification of pathogenicity.

The exomes of 69 FH patients were analyzed, as described (Fig 1). Of the 23 patients with mutations in 3 FH-linked genes, known causal mutations were found in 18 patients (Table 2). Among them, 15 patients were identified as having pathogenic mutations in *LDLR*. Specifically, two patients had the p.P685L mutation and four patients had either the p.E228X or p.E228K mutation. During preliminary screening by Sanger sequencing prior to WES, we found three more patients with the p.P685L mutation, which suggests the presence of potential mutational hotspots in Korean FH cases. In addition, both the p.R257W and p.D589N homozygous mutations were found in one patient. This patient had an LDL-C level of 340 mg/dL, which was relatively higher than those of patients with only one heterozygous mutation. Two patients harbored the p.R3527Q mutation in *APOB*, the most common causal variation of *APOB* in other populations. [18] Lastly, one patient had the p.E32K mutation in *PCSK9*. [19, 20]

**Table 2:** Known pathogenic mutations in three FH-linked genes (n = 69).

Gene	Genomic coordinate	Nucleotide change*	Mutation type	Amino acid change	Affected patients (frequency)
*LDLR*	chr19:11213417	c.268G>A	Missense	p.D90N	1 (0.014)
*LDLR*	chr19:11216000	c.418G>A	Missense	p.E140K	1 (0.014)
*LDLR*	chr19:11216101	c.519C>G	Missense	p.C173W	1 (0.014)
*LDLR*	chr19:11216243	c.661G>A	Missense	p.D221N	1 (0.014)
*LDLR*	chr19:11216264	c.682G>T	Nonsense	p.E228X	3 (0.043)
*LDLR*	chr19:11216264	c.682G>A	Missense	p.E228K	1 (0.014)
*LDLR*	chr19:11217315	c.769C>T	Missense	p.R257W	1^†^ (0.014)
*LDLR*	chr19:11227594	c.1765G>A	Missense	p.D589N	
*LDLR*	chr19:11224013	c. 1246C>T	Missense	p.R416W	1 (0.014)
*LDLR*	chr19:11226885	c.1702C>G	Missense	p.L568V	1 (0.014)
*LDLR*	chr19:11231112	c. 2054C>T	Missense	p.P685L	2^‡^ (0.028)
*LDLR*	chr19:11221326	c.941-2A>G	Splicing, acceptor site (exon6-2nt)	Frameshift	1 (0.014)
*LDLR*	chr19:11222188	c.1061-2A>G	Splicing, acceptor site (exon7-2nt)	Frameshift	1 (0.014)
*APOB*	chr2:21229160	c.10580C>T	Missense	p.R3527Q	2 (0.028)
*PCSK9*	chr1:55505604	c.94G>A	Missense	p.E32K	1 (0.014)

*Nucleotide location number was assigned according to the low-density lipoprotein receptor (*LDLR*; NM_000527), apolipoprotein B (*APOB*; NM_000384), and proprotein convertase subtilisin/kexin type 9 (*PCSK9*; NM_174936) mRNA sequences.

^†^A patient (P49) with p.R257W (homozygote) and p.D589N (homozygote).

^‡^Screening the remaining cohort by Sanger sequencing identified three more patients with p.P685L.

Variants were characterized in published studies and validated in the present study by Sanger sequencing.

### Discovery and validation of novel mutations in the three FH-linked genes

**Fig 2:**
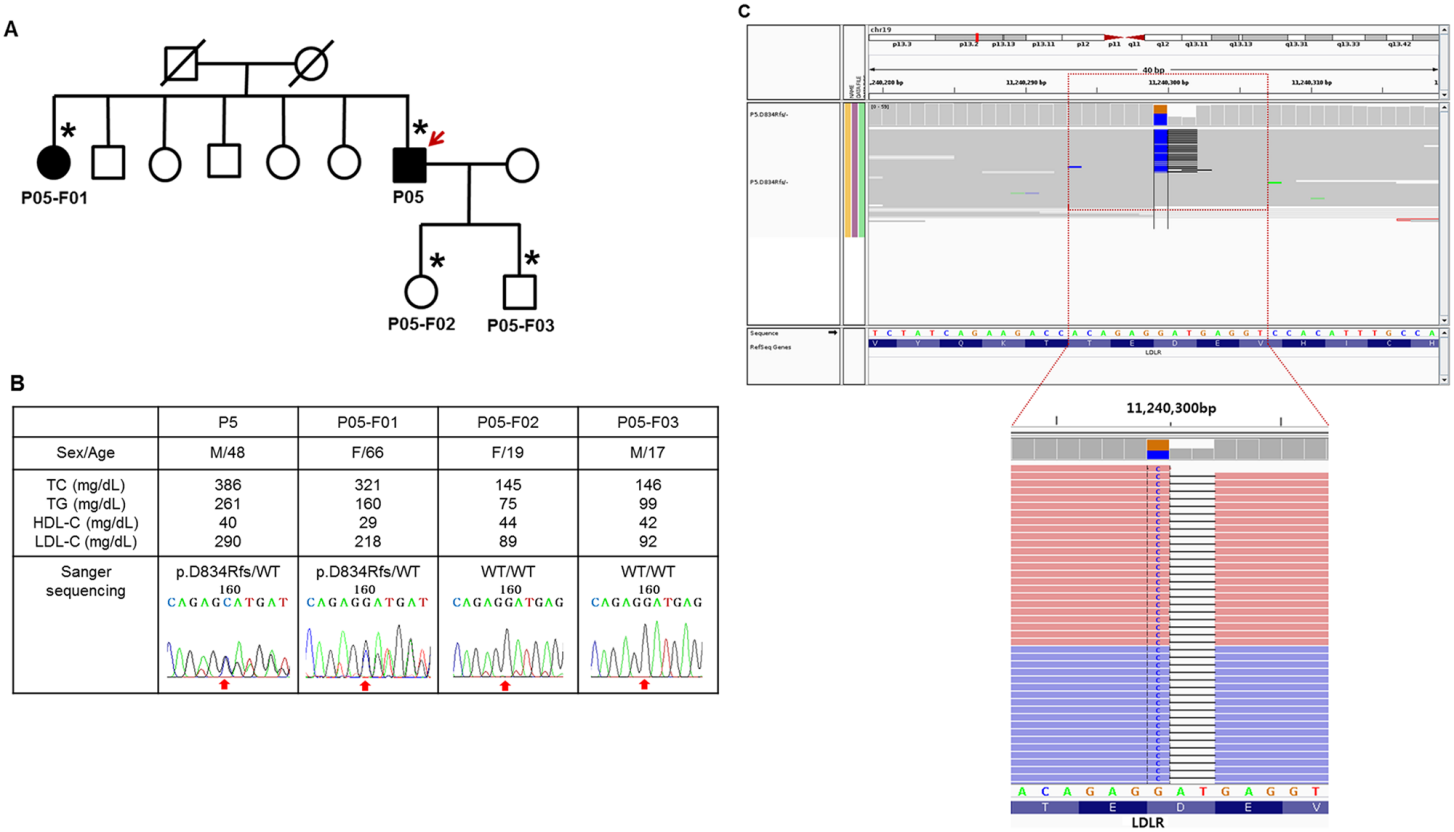
Pedigree analysis of a patient with *LDLR* p.D834Rfs/- mutation. (A) A simplified pedigree of the P05 family. The upper right arrow indicates the proband; squares indicate males, and circles indicate females. Open and filled symbols indicate unaffected and affected individuals, respectively. Asterisks indicate family members who underwent clinical examinations and molecular analyses. WT refers wild-type. (B) Clinical examination data and sequencing chromatograms. Vertical arrows indicate the mutation site. (C) Integrative Genomics Viewer screenshot of p.D834Rfs/-. Sequencing reads show that a single nucleotide substitution (G>C) and frameshift deletion (AT/-) occurred at the cis position.

In addition to known mutations, novel disruptive mutations were detected in three FH patients. Two novel mutations were predicted to disrupt the *LDLR* gene due to a frame shift (Table 3). Neither mutation was observed in any control exome data or public databases. The 13-nt deletion mutation (c.320_332delGACGTGCTCCCAG) was thought to be a pathogenic due to frame shift disruptions that introduced a premature stop codon. The p.D834Rfs mutation resulted from two concurrent mutations (c.2500_2502delGAT and c.2500insC) consecutively at the cis-position and was confirmed as a D834Rfs/- heterozygous mutation, using the Integrative Genomics Viewer (Fig 2). Though it occurred at a relatively posterior position among the 860 coding region of *LDLR*, we could still confirm p.D834Rfs as causal variant that co-segregated within the corresponding family (P05; Fig 2). One variant in *PCSK9*, p.R215C, was identified within an evolutionarily conserved loop within the catalytic domain. [21] Considering that gain-of-function mutations within this loop have been confirmed, [22, 23] p.R215C is likely to be a gain-of-function variant.

**Table 3:** Novel pathogenic mutations in three FH-linked genes (n = 69).

Detailed information of novel mutations						Pathogenicity^†^			
Gene	Genomic coordinate	Nucleotide change*	Mutation type	Amino acid change	Affected patients (frequency)	MAF in Korean controls (n = 390)	Frequency in public databases^‡^	Polyphen-2 prediction (probability)	SIFT prediction (score)
*LDLR*	chr19:11215902–11215914	c.321_333del-GACGTGCTCCCAG	Frameshift deletion	p.C109Sfs^§^	1 (0.014)	0	Novel	NA	NA
*LDLR*	chr19:11240299	c.2500_2502del-GATinsC^**||**^	Frameshift deletion/insertion	p.D834Rfs	1 (0.014)	0	Novel	NA	NA
*PCSK9*	chr1:55518070	c.643C>T	Missense	p.R215C	1 (0.014)	0	Novel^**#**^	Probably damaging (1)	Damaging (0.008)

*Nucleotide location number was assigned according to the low-density lipoprotein receptor (*LDLR*; NM_000527) and proprotein convertase subtilisin/kexin type 9 (*PCSK9*; NM_174936) mRNA sequences.

^†^Prediction for frameshift mutations of *LDLR* is not available from the Polyphen-2 and SIFT algorithms and is not marked.

^‡^Public databases include the 1000 Genomes Project, dbSNP135, and NHLBI GO Exome Sequencing Project.

^§^The frameshift mutation changes the cysteine at position 109, as four nucleotides after the deletion compensate for the frameshift effect until threonine (108).

^||^The replacement of nucleotides 2500 to 2502 (GAT) occurred by ‘C’ at the cis position.

^#^The p.R215H (c.644G>A) is a gain-of-function mutation in the catalytic domain of *PCSK9*. [22, 23] Variants were validated by Sanger sequencing.

NA: Not available.

### Copy number analysis and validation

**Fig 3:**
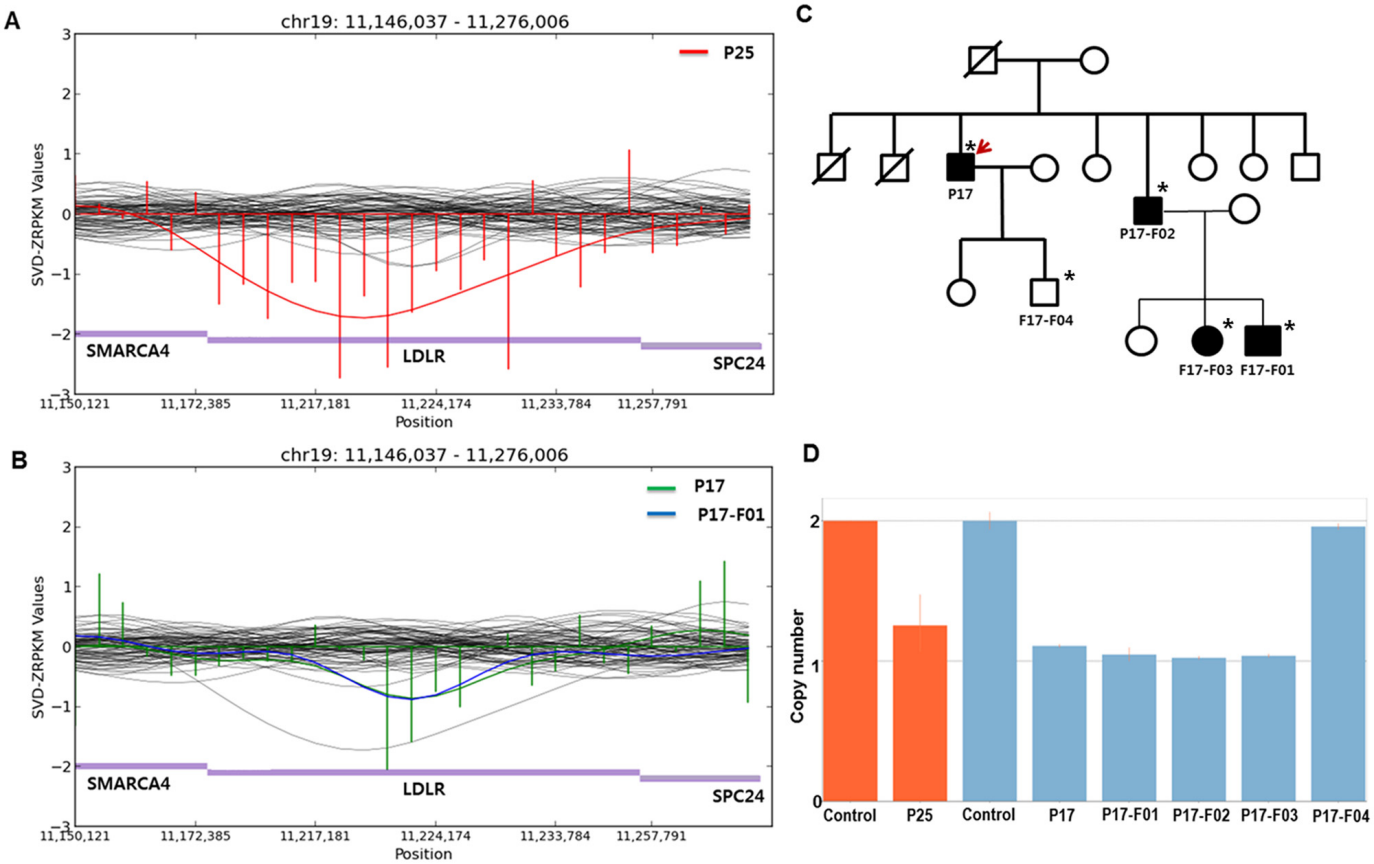
Copy number variation (CNV) detection in *LDLR*. SVD-ZRPKM values were used to detect CNVs by the CoNIFER algorithm and were calculated by transforming reads per kilobase per million values into standardized z-scores, based on the mean and standard deviation across all analyzed exomes. (A) The SVD-ZRPKM regional plot of the P25 patient with a large copy number deletion in *LDLR*. (B) The SVD-ZRPKM regional plot of the P17 patient and family member (P17-F01) with an inherited copy number deletion in *LDLR*. Green and blue indicate SVD-ZRPKM values of P17 and P17-F01, respectively. Values are plotted based on P17. (C) Pedigree of the P17 patient with CNV. The upper right arrow indicates the proband; squares indicate males, and circles indicate females. Open and filled symbols indicate unaffected and affected individuals, respectively. Asterisks indicate family members who underwent clinical examinations and CNV analyses. (D) TaqMan Copy Number Assay for P25, P17, and family members of P17. Red indicates the assay for P25 by probe #1 within intron 5; blue indicates the assay for P17 and other members by probe #2 (overlapped from intron 10 to exon 11). The assay was performed in duplicate and repeated. Results were plotted by CopyCaller software v.2.0.

In the CNV analysis, novel copy number deletions were detected in two patients. A fragment spanning from exon 1 to exon 12 of *LDLR* was inferred as being lost in one patient (P25) by the CoNIFER algorithm (Fig 3A). [17] The copy number was measured to be half of that of the control by Taqman copy number assay, which detected intron 5 (Fig 3D). Another copy number deletion ranging from exon 8 to exon 12 of *LDLR* was detected and validated by Taqman assay (Fig 3B and 3D). Notably, the copy number loss co-segregated in other affected family members as well, indicating the deletion to be the causal alteration for FH in the corresponding family (P17; Fig 3C and 3D).

## Discussion

In recent years, NGS has been applied in studies on FH and demonstrated as an efficient tool to identify causing mutations in known and novel genes. [10, 24] The current study utilized WES to identify FH-causing mutations in Korean FH cases and presented the values of WES in the diagnosis of FH. The exome-based diagnosis was comprehensive, as it confirmed genetic variations that were not merely limited to SNVs and included small insertions or deletions and copy number variations. In fact, we confirmed 23 causative mutations in 3 known FH genes, including 2 copy number deletions, through WES-based genetic testing. Importantly, this suggests that newly generated DNA sequences can be used for the discovery and genetic diagnosis of novel FH-causing genes without the need for additional costs. The WES data in genetically undiagnosed patients from this study will be used for further analyses.

A DNA test, along with family screening, is confirmatory and clinically relevant, especially for individuals with borderline-high serum LDL-C levels. Furthermore, the co-segregation analysis of variants in families provides strong evidence of the pathogenicity of undefined variants in genetic testing. During initial exploratory screening through WES analysis, we found a total of four novel variants in *LDLR* with uncertain pathogenicity. Among them, only two variants were found to be causative, based on *in silico* and family co-segregation analyses. In the case of *APOB*, we found several novel variants with uncertain pathogenicity located outside of LDLR-binding regions. All variants were ruled out by family co-segregation analysis.

Of the FH cases with confirmed mutations, one patient was homozygous for both p.R257W and p.D589N mutations in *LDLR* and had relatively higher LDL-C levels than those of patients with only one heterozygous mutation. The exact same double homozygous mutation has been previously described in one Taiwanese patient with FH. [25] Both patients exhibited tendon xanthomas without presenting typical homozygous phenotypes. Therefore, we hypothesize that this mutation does not fully abolish LDLR activity. However, further functional studies are needed.

Cholesterol levels in Asian FH patients are lower than those of Western patients. [26] The mean LDL-C of patients in this study was 230 mg/dL, which was similar to that of Japanese patients [27] but lower than those of Chinese or Taiwanese patients. [25, 28] In our study, the mutation carriers had significantly higher LDL-C levels. This finding is in accordance with prior reports and indicates that a higher level of LDL-C is an important characteristic of mutation-positive patients. [29, 30] The proportion of male was higher in mutation carriers. The mean age of females was 57 years and higher than that of males. Post-menopausal females can be subject to nonspecific elevation of blood cholesterol, which may have increased the number of false-positive cases in our study. [31] The prevalence of coronary artery disease in our cohort was 36%, and this can vary widely in Asian populations. [26] There was no difference in the prevalence between patients with and without mutations.

The mutation detection rate in the 3 known FH genes was 33% in our study, which is lower than those of recent reports. [25, 26, 32, 33] One possible reason is that the majority of subjects were initially classified as possible FH patients whose mutation rates were lower than those of definite FH patients. In addition, the low-sequencing coverage of known FH-causing genes, especially *PCSK9*, may have lowered the mutation detection rate (S1 Fig). Therefore, we cannot rule out the possibility of undetected mutations in these regions. For the use of WES in the genetic diagnosis of FH, further efforts are needed to improve coverage for regions with insufficient coverage in FH-causing genes. As sequencing costs are dropping, the cost for WES becomes quite low. As a result, high-depth WES can achieve high coverage in FH-targeted genes and display better diagnostic performance.

There are several points that haven’t fully covered for the genetic diagnosis in FH cases without defined mutations. Originally, we set the values of WES in an attempt to identify novel FH-causing genes, going beyond the confirmatory exome-based diagnosis of FH. However, when applying existing statistical methods to reveal novel FH-causing genes, we found that the tests were underpowered and could not fully verify the causality of putative genes. These were mainly due to the small sample size. Furthermore, we could not define polygenic FH [34, 35] through WES by calculating LDL-C gene scores, as most of these scores were calculated according to the number of SNPs that occurred mostly in introns. Further research is needed to evaluate polygenic cause in Korean FH cases without defined mutations.

In summary, we identified 23 mutations in known FH genes (19 in *LDLR*, two in *APOB*, and two in *PCSK9*) using WES in 69 FH patients who met Simon Broome criteria with definite family history. We also identified three new causative mutations: two frame shift deletions in *LDLR* and one mutation in *PCSK9*.

## Supporting Information

S1 Fig Summary statistics of 3 FH genes. Variant callable portion was defined as locus covered at least of 8× fold coverage by sequencing.
(TIF)

S1 Table Summary statistics of whole-exome sequencing data. (DOCX)

S2 Table Detailed information of TaqMan Copy Number Assay. (DOCX)
