# Supplementary figures and images for: Genetic Testing of Korean Familial Hypercholesterolemia Using Whole-Exome Sequencing
Source: PLoS One. 2015 May 11;10(5):e0126706. doi: 10.1371/journal.pone.0126706 (PMC4427254; doi:10.1371/journal.pone.0126706)

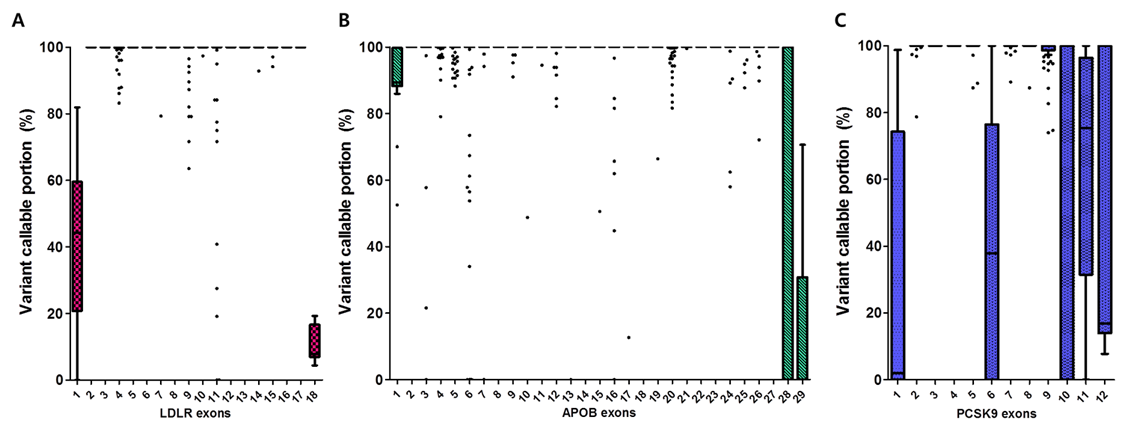

Supplement: S1 Fig — Variant callable portion was defined as locus covered at least of 8× fold coverage by sequencing. (TIF) [file pone.0126706.s001.tif]
