# Supplementary material for: Genetic Testing of Korean Familial Hypercholesterolemia Using Whole-Exome Sequencing
Source: PLoS One. 2015 May 11;10(5):e0126706. doi: 10.1371/journal.pone.0126706 (PMC4427254; doi:10.1371/journal.pone.0126706)
Supplement: S1 Table — (DOCX) [file pone.0126706.s002.docx]

**S1 Table. Summary statistics of whole-exome sequencing data**

|  |  |  | Median Coverage (%) | | |
| --- | --- | --- | --- | --- | --- |
|  |  | Median depth (x) | Over 1x | Over 8x | Over 20x |
| FH patients  (n=69) | SureSelect All Exon 50Mb (n=44) | 49.59 | 95.71 | 88.36 | 75.62 |
|  | SureSelect All Exon V4+UTRs (n=25) | 62.96 | 99.37 | 96.13 | 83.46 |
| Healthy control  (n=390) | SureSelect All Exon 50Mb (n=145) | 60.31 | 92.16 | 84.16 | 74.96 |
|  | Truseq (n=118) | 34.40 | 66.13 | 53.44 | 46.36 |
|  | SureSelect All Exon V4+UTRs (n=127) | 92.68 | 99.48 | 97.99 | 92.15 |
