# Supplementary material for: Genetic Testing of Korean Familial Hypercholesterolemia Using Whole-Exome Sequencing
Source: PLoS One. 2015 May 11;10(5):e0126706. doi: 10.1371/journal.pone.0126706 (PMC4427254; doi:10.1371/journal.pone.0126706)
Supplement: S2 Table — (DOCX) [file pone.0126706.s003.docx]

**S2 Table. Detailed information of TaqMan Copy Number Assay**

| Gene | Assay ID | Assay Location  (NCBI Build 37) | Assay cytoband | Assay  gene Location |
| --- | --- | --- | --- | --- |
| *LDLR* | Hs07151140_cn | Chr19:11218010 | 19p13.2b | Within Intron 5 |
| *LDLR* | Hs07156441_cn | Chr19:11226702 | 19p13.2b | Overlaps Intron 10 - Exon 11 |
